# Supplementary material for: Heat adaptation of phage T7 under an extended genetic code
Source: Virus Evol. 2021 Dec 1;7(2):veab100. doi: 10.1093/ve/veab100 (PMC8923235; doi:10.1093/ve/veab100)
Supplement: veab100_Supp [file veab100_supp.zip › Sequencing_Analysis.zip!/Sequencing_Analysis/A3/evidence/RA_18.html]

BRESEQ :: Evidence


| Read alignment evidence... | | | | | | | | | | | |
| --- | --- | --- | --- | --- | --- | --- | --- | --- | --- | --- | --- |
|  | seq id | position | | ref | new | freq | score (cons/poly) | reads | annotation | genes | product |
| \* | NC\_001604.1 | 1,242 | 1 | . | G | 27.8% | 3018.8 / inf | 1847 | intergenic (–/–) | *–/–* | –/– |
| Reads supporting (aligned to +/- strand):  **ref** base . (631/693);  **new** base G (263/247);  **total** (899/948) | | | | | | | | | | | |
| Fisher's exact test for biased strand distribution *p*-value = 1.44e-01 | | | | | | | | | | | |
| Kolmogorov-Smirnov test that lower quality scores support variant *p*-value = 2.51e-02 | | | | | | | | | | | |

Only 100 of 1868 total aligned reads displayed.  

`GACTTACAACAACGTTTTCGACCACGCTTACGAAATGCTGAAAGAAAACATCCGTTATGATGACATCCGTGACACTGATGACCTGCACGATGCTATTCACATGGCTGCCGATAATGCAGTTCCGCACTACTACGCTGACATCTTTAGCGTAATGGCAAGTGAGGGCATTGACCTTGAGTTCGAAGACTCTGGTCTGATGCCTGACACCAAGGACGTAATCCGCATCCTGCAAGCGCGTATCTATGAGCAATTAACGATTGACCTCTGGGAAGACGCAGAAGACTTGCTCAATGAATACTTG‑‑‑‑‑‑‑‑‑GAG‑‑‑‑‑‑‑‑‑GAAGTC‑‑‑‑‑‑‑‑‑GAGGAGTACGAGGAGGATGAAGAGTAATGTCTACTACCAACGTGCAATACGGTCTGACCGCTCAAACTGTACTTTTCTATAGCGACATGGTGCGCTGTGGCTTTAACTGGTCACTCGCAATGGCACAGCTCAAAGAACTGTACGAAAACAACAAGGCAATAGCTTTAGAATCTGCTGAGTGATAGACTCAAGGTCGCTCCTAGCGAGTGGCCTTTATGATTATCACTTTACTTATGAGGGAGTAATGTATATGCTTACTATCGGTCTACTCACCGCTCTAGGTCTAG  >  NC_001604.1/942‑1538`  

                                                                                                                                                                                                                                                                                                             `|`                                                                                                                                                                                                                                                                                                                                    
 `aCTTACAACAACGTTTTCGACCACGCTTACGAAATGCTGAAAGAAAACATCCGTTATGATGACATCCGTGACACTGATGACCTGCACGATGCTATTCACATGGCTGCCGATAATGCAGTTCCGCACTACTACGCTGACATCTTTAGCGTAATGGCAAGTGAGGGCATTGACCTTGAGTTCGAAGACTCTGGTCTGATGCCTGACACCAAGGACGTAATCCGCATCCTGCAAGCGCGTATCTATGAGCAATTAACGATTGACCTCTGGGAAGACGCAGAAGACTTGCTCAATGAATACTTG‑‑‑‑‑‑‑‑‑G                                                                                                                                                                                                                                                                                                                           <  1:59799‑M1/301‑1 (MQ=255)`  
    `tACACCAACGTTTTCGACCACGCTTACGAAATGCTGAAAGAAAACATCCGTTATGATGACATCCGTGACACTGATGACCTGCACGATGCTATTCACATTGCTGCCGATAATTCAGTTCCGCACTACTACGCTGACATCTTTAGCGTAATGGCAAGTGAGGGCACTGATCTTGAGTTCGAAGACTCTGGTCTGATGCCTGACACCAAGGACGTAATCCGCATCCTGCAAGCGCGTAACTATGAGCAATTAACGATTGACCTCTGGGAAGACGCAGAAGACTTGCTCAATGAATACTTG‑‑‑‑‑‑‑‑‑GAG‑‑‑‑‑‑‑‑‑G                                                                                                                                                                                                                                                                                                               <  1:137341‑M1/301‑1 (MQ=255)`  
                         `taagagacagtGCTGAAAGAAAACATCCGTTATGATGACATCCGTGACACTGATGACCTGCACGATGCTATTCACATGGCTGCCGATAATGCAGTTCCGCACTACTACGCTGACATCTTTAGCGTAATGGCAAGTGAGGGCATTGACCTTGAGTTCGAAGACTCTGGTCTGATGCCTGACACCAAGGACGTAATCCGCATCCTGCAAGCGCGTATCTATGAGCAATTAACGATTGACCTCTGGGAAGACGCAGAAGACTTGCTCAATGAATACTTG‑‑‑‑‑‑‑‑‑GAG‑‑‑‑‑‑‑‑‑GAAGTC‑‑‑‑‑‑‑‑‑gaggaagtcgcacgac                                                                                                                                                                                                                                                                                 <  1:264112/291‑12 (MQ=255)`  
                         `gCTTACGAAATGCTGAAAGAAAACATCCGTTATGATGACATCCGTGACACTGATGACCTGCACGAAGCTATTCACATGGCTGCCGATAATGCAGTTCCGCACTACTACGCTGACATCTTTAGCGTAATGGCAAGTGAGGGCATTGACCTTGAGTTCGAAGACTCTGGTCTGATGCCTGACACCAAGGACGTAATCCGCATCCTGCAAGCGCGTATCTATGAGCAATTAACGATTGACCTCTGGGAAGACGCAGAAGACTTGCTCAATGAATACTTG‑‑‑‑‑‑‑‑‑GAG‑‑‑‑‑‑‑‑‑GAAGTC‑‑‑‑‑‑‑‑‑gaggaagtcgcacgac                                                                                                                                                                                                                                                                                 <  1:103930/301‑12 (MQ=255)`  
                          `cTTACGAAATGCTGAAAGTAAACATCCGTTATGATGACATCCGTGACACTGATGACCTGCACGATGCTATTCACATGGCTGCCGATAATGCAGTTCCGCACTACTACGCTGACATCTTTAGCGTAATGGCAAGTGAGGGCATTGACCTTGAGTTCGAAGACTCTGGTCTGATGCCTGACACCAAGGACGTAATCCGCATCCTGCAAGCGCGTATCTATGAGCAATTAACGATTGACCTCTGGGAAGACGCAGAAGACTTGCTCAATGAATACTTG‑‑‑‑‑‑‑‑‑GAG‑‑‑‑‑‑‑‑‑GActgtctcttatacacacctcc                                                                                                                                                                                                                                                                                         >  1:318492‑M1/1‑280 (MQ=255)`  
                          `attaCGAAATGCTGAAAGAAAAAATCCGGTATGATGACATCCGTGAAACTGATGACCTGCACGATGCGATTCACATGGCTGCCGATAATGCAGTTCCGCACTACTACGCTGACATCTTTAGCGTAATGGCAAGTGAGGGCATTGACATTGAGTTCGAAGACTCTGGTCTGATGCCTGACACCAAGGACGTAATCCGCATCCTGCAAGCGCGTATCTATGAGCAATTAACGATTGACCTCTGGGAAGACGCAGAAGAATTGCTCAATGAATACTTG‑‑‑‑‑‑‑‑‑GAG‑‑‑‑‑‑‑‑‑GAAGTC‑‑‑‑‑‑‑‑‑Gcacgacagaaagaaat                                                                                                                                                                                                                                                                                <  1:342136‑M1/300‑17 (MQ=255)`  
                                `aaaTGCTGAAAGAAAACATCCGTTATGATGACATCCGTGACACTGATGACCTGCACGATGCTATTCACATGGCTGCCGATAATGCAGTTCCGCACTACTACGCTGACATCTTTAGCGTAATGGCAAGTGAGGGCATTGACCTTGAGTTCGAAGACTCTGGTCTGATGCCTGACACCAAGGACGTAATCCGCATCCTGCAAGCGCGTATCTATGAGCAATTAACGATTGACCTCTGGGAAGACGCAGAAGACTTGCTCAATGAATACTTG‑‑‑‑‑‑‑‑‑GAG‑‑‑‑‑‑‑‑‑GAAGTC‑‑‑‑‑‑‑‑‑gaggaagtcgcacgacagaaaga                                                                                                                                                                                                                                                                          <  1:241450/301‑19 (MQ=255)`  
                                `aaaTGCTGAAAGAAAACATCCGTTATGATGACATCCGTGACACTGATGACCTGCACGATGCTATTCACATGGCTGCCGATAATGCAGTTCCGCACTACTACGCTGACATCTTTAGCGTAATGGAAAGTGAGGGCATTGACCTTGAGTTCGAAGACTCTGGTCTGATGCCTGACACCAAGGACGTAATCCGCATCCTGCAAGCGCGTATCTATGAGCAATTAACGATTGACCTCTGGGAAGACGCAGAAGACTTGCTCAATGAATACTTG‑‑‑‑‑‑‑‑‑GAG‑‑‑‑‑‑‑‑‑GAAGTC‑‑‑‑‑‑‑‑‑gaggaagtcgcacgacagaaaga                                                                                                                                                                                                                                                                          <  1:256595/301‑19 (MQ=255)`  
                                                     `gTTATGATGACATCCGTGACACTGATGACCTGCACGATGCTATTCACATGGCTGCCGATAATGCAGTTCCGCACTACTACGCTGACATCTTTAGCGTAATGGCAAGTGAGGGCATTGACCTTGAGTTCGAAGACTCTGGTCTGATGCCTGACACCAAGGACGTAATCCGCATCCTGCAAGCGCGTATCTATGAGCAATTAACGATTGACCTCTGGGAAGACGCAGAAGACTTGCTCAATGAATACTTG‑‑‑‑‑‑‑‑‑GAG‑‑‑‑‑‑‑‑‑GAAGTC‑‑‑‑‑‑‑‑‑Gcacgacagaaagaaattgacagcgctaaggcccgtaaagaacg                                                                                                                                                                                                                                                     >  1:207744‑M1/1‑258 (MQ=255)`  
                                                          `gatgaCATCCGTGACACTGATGACCTGCACGATGCTATTCACATGGCTGCCGATAATGCAGTTCCGCACTACTACGCTGACATCTTTAGCGTAATGGCAAGTGAGGGCATTGACCTTGAGTTCGAAGACTCTGGTCTGATGCCTGACACCAAGGACGTAATCCGCATCCTGCAAGCGCGTATCTATGAGCAATTAACGATTGACCTCTGGGAAGACGCAGAAGACTTGCTCAATGAATACTTG‑‑‑‑‑‑‑‑‑GAG‑‑‑‑‑‑‑‑‑GAAGTC‑‑‑‑‑‑‑‑‑Gcacgacagaaagaaattgaccgcgctaaggcccgtaaagaacgtcacg                                                                                                                                                                                                                                                <  1:16881‑M1/301‑49 (MQ=255)`  
                                                             `aacATCCGTGACACTTAGGACCTGCACGATGCTATTCAAATGGCTGCAGATAAGGCAGTTCCGAACTACTACGCGGACATAGTGAGCGGAATGGCAAGTGAGGGCATTGAACTTGAGTTCGAAGACTCTGGTCTGATGCCTGACACCAAGGACGTAATCCGCATCCTGCAAGCGCGTATCTATGAGCAATTAACGATTGACCTCTGGGAAGACGCAGAAGACTTGCTCAATGAATACTTGGAGGAAGTCGAG‑‑‑‑‑‑‑‑‑GAAGTC‑‑‑‑‑‑‑‑‑Gcacgacagaaagaaattgaccgcgctaaggcccgtaaagaac                                                                                                                                                                                                                                                      <  1:70705‑M1/300‑43 (MQ=255)`  
                                                                        `acctgATGACCTGCACGATGCTATTAACATGGCTGCCGATAATGCAGTTCCGCACTACTACGCTGACATCTTTAGCGTAATGGCAAGTGAGGGCATTGACCTTGAGTTCGAAGACTCTGGTCTGATGCCTGACACCAAGGACGTAATCAGCATCCTGCAAGCGCGTATCTATGAGCAATTAACGATTGACCTCTGGGAAGACGCAGAAGACTTGCTCAATGAATACTTGGAGGAAGTAGAG‑‑‑‑‑‑‑‑‑GAAGTC‑‑‑‑‑‑‑‑‑Gcacgacagaaagaaattgaccgcgctaaggcccgtaaagaacgtcacgagggg                                                                                                                                                                                                                                           <  1:39489‑M1/299‑54 (MQ=255)`  
                                                                         `acggatgacatGCAAGCTGCTATTCACATGGCAGCCTATAATGCAGTTCCGCACAGCTACGCTGACATCTTTAGCTTAATGGCAAGTGAGGGCATTGACCTTGAGTTAGAAGACTCGGGTCTGATGCCTGACGCCAAGGACGTAATCCGCATCCTGCAAGCGCGTATCTATGAGCAATTAACGAGTGACCTCTGGGAAGTCGCAGAAAACTTGCTCAATGAATACTGG‑‑‑‑‑‑‑‑‑GGG‑‑‑‑‑‑‑‑‑GAAGTC‑‑‑‑‑‑‑‑‑Gaacgaaagaaagaaattgaccgcgctaaggcccgtaaagaacgtcacgaggggcgcatagagg                                                                                                                                                                                                                                 <  1:53724‑M1/298‑64 (MQ=255)`  
                                                                               `gaCCTGCACGATGCTATTCACATGGCTGCCTATAATGCAGTTCCGCACTACTACGCTGACATCTTTAGCGTAATGGCAAGTGAGGGCATTGACCTTGAGTTCGAAGACTCTGGTCTGATGCCTGACACCAAGGACGTAATCCGCATCCTGCAAGCGCGTATCTATGAGCAATTAACGATTGACCTCTGGGAAGACGCAGAAGACTTGCTCAATGAATACTTG‑‑‑‑‑‑‑‑‑GAG‑‑‑‑‑‑‑‑‑GAAGTC‑‑‑‑‑‑‑‑‑Gcacgacagaaagaaattgaccgcgctaaggcccgtaaagaacgtcacgaggggcgcttagaggcacgca                                                                                                                                                                                                                           >  1:48408‑M1/1‑232 (MQ=255)`  
                                                                               `gaCCTGCACGATGCTATTCACATGGCTGCCGATAATGCAGTTCCGCACTACTACGCTGACATCTTTAGCGTAATGGCAAGTGAGGGCATTGACCTTGAGTTCGAAGACTCTGGTCTGATGCCTGACACCAAGGACGTAATCCGCATCCTGCAAGCGCGTATCTATGAGCAATTAACGATTGACCTCTGGGAAGACGCAGAAGACTTGCTCAATGAATACTTGGAGGAAGTCGAG‑‑‑‑‑‑‑‑‑GAAGTC‑‑‑‑‑‑‑‑‑Gcacgacagaaagaaattgaccgcgctaaggcccgtaaagaacgtcacgaggggcgcttag                                                                                                                                                                                                                                    >  1:267034‑M1/1‑241 (MQ=255)`  
                                                                                 `ttataagagacagattcacatggctgcCGATAATGCAGTTCCGCACTACTACGCTGACATCTTTAGCGTAATGGCAAGTGAGGGCATTGACCTTGAGTTCGAAGACTCTGGTCTGATGCCTGACACCAAGGACGTAATCCGCATCCTGCAAGCGCGTATCTATGAGCAATTAACGATTGACCTCTGGGAAGACGCAGAAGACTTGCTCAATGAATACTTG‑‑‑‑‑‑‑‑‑GAG‑‑‑‑‑‑‑‑‑GAAGTC‑‑‑‑‑‑‑‑‑Gcacgacagaaagaaattgaccgcgctaaggcccgtaaagaacgtcacgaggggcgcttagaggcacgcaga                                                                                                                                                                                                                         <  1:86755‑M1/288‑72 (MQ=255)`  
                                                                                 `ccTGCACGATGCTCTTCACATGGCTGCCGATAATGCAGTTCCGCACTACTACGCTGACATCTTTAGCGTAATGGCAAGTGAGGGCATTGACCTTGAGTTCGAAGACTCTGGTCTGATGCCTGACACCAAGGACGTAATCCGCATCCTGCAAGCGCGTATCTATGAGCAATTAACGATTGACCTCTTGGAAGACGCAGAAGACTTGCTCAATGAATACTTG‑‑‑‑‑‑‑‑‑GAG‑‑‑‑‑‑‑‑‑GAAGTC‑‑‑‑‑‑‑‑‑Gcacgacagaaagaaattgaccgcgctaaggcccgtaaagaacgtcacgacgggcgcttagaggcacgccga                                                                                                                                                                                                                         >  1:64443‑M1/1‑230 (MQ=255)`  
                                                                                  `cTGCACGATGCTATTCACATGGCTGCCGATAATGCAGTTCCGCACTACTACGCTGACATCTTTAGCGTAATGGCAAGTGAGGGCATTGACCTTGAGTTCGAAGACTCTGGTCTGATGCCTGCCACCAAGGACGTAATCCGCATCATGCAAGCGCGTATCTATGAGCAATTAACGATTGACCTCTGGGAAGACGCAGAAGACTTGCTCAATGAATACTTGGAGGAAGTCGAG‑‑‑‑‑‑‑‑‑GAAGTC‑‑‑‑‑‑‑‑‑Gcacgacagaaagaaattgaccgcgctaaggcccgtaaagaacgtcacgaggggcacttagagg                                                                                                                                                                                                                                 <  1:96687‑M1/301‑64 (MQ=255)`  
                                                                                  `cTGCACGATGCTATTCACATGGCTGCCGATAATGCAGTTCCGCACTACTACGCTGACATCTTTAGCGTAATGGCAAGTGAGGGCATTGACCTTGAGTTCGAAGACTCTGGTCTGATGCCTGACACCAAGGACGTAATCCGCATCCTGCAAGCGCGTATCTATGAGCAATTAACGATTGACCTCTGGGAAGACGCAGAAGACTTGCTCAATGAATACTTGGAGGAAGTCGAG‑‑‑‑‑‑‑‑‑GAAGTC‑‑‑‑‑‑‑‑‑Gcacgacagaaagaaattgaccgcgctaaggcccgtaaagaacgtcacgaggggcgcttagagg                                                                                                                                                                                                                                 <  1:161277‑M1/301‑64 (MQ=255)`  
                                                                                    `gCACGATGCTATTCACATGGCTGCCGATAATGCAGTTCCGCACTACTACGCTGACATCTTTAGCGTAATGGCAAGTGAGGGCATTGACCTTGAGTTCGAAGACTCTGGTCTGATGCCTGACACCAAGGACGTAATCCGCATCCTGCAAGCGCGTATCTATGAGCAATTAACGATTGACCTCTGGGAAGACGCAGAATACTTGCTAAATGAATACTTGGAGGAAGTCGAG‑‑‑‑‑‑‑‑‑GAAGTC‑‑‑‑‑‑‑‑‑Gcacgacagaaagaatttgaccgcgctaaggcccgtaaagaacgtcacgaggggcgcttagaggca                                                                                                                                                                                                                               >  1:325248‑M1/1‑236 (MQ=255)`  
                                                                                        `agacagattcacaTGGCTGCCGATAATGCAGTTCCGCACTACTACGCTGACATCTTTAGCGTAATGGCAAGTGAGGGCATTGACCTTGAGTTCGAAGACTCTGGTCTGATGCCTGACACCAAGGACGTAATCCGCATCCTGCAAGCGCGTATCTATGAGCAATTAACGATTGACCTCTGGGAAGACGCAGAAGACTTGCTCAATGAATACTTG‑‑‑‑‑‑‑‑‑GAG‑‑‑‑‑‑‑‑‑GAAGTA‑‑‑‑‑‑‑‑‑Gcacgacagaaagaaattgaccgcgctaaggcccgtaaagaacgtcacgaggggcgcttagaggcacgcagattcaaac                                                                                                                                                                                                                  <  1:247975‑M1/295‑79 (MQ=255)`  
                                                                                        `aagagacagcacatggctgccgATAATGCAGTTCCGCACTACTACGCTGACATCTTTAGCGTAATGGCAAGTGAGGGCATTGACCTTGAGTTCGAAGACTCTGGGCTGATGCCTGACACCAAGGACGTAATCCGCATCATGCAAGCGCGTATCTATGAGCAATGAACGATTGACCTCTGGGAAGACGCAGAAGACTTGCTCAATGAATACTTG‑‑‑‑‑‑‑‑‑GAG‑‑‑‑‑‑‑‑‑GAAGTC‑‑‑‑‑‑‑‑‑Gcacgacagaaagaaattgaccgcgctaaggcccgtaaagaacgtcacgaggggcacttagaggcacgcagattcaaac                                                                                                                                                                                                                  <  1:141848‑M1/292‑79 (MQ=255)`  
                                                                                              `aTTCACATGGCTGCCGATAATGCAGTTCCGCACTACTACGCTGACATCTTTAGCGTAATGGCAAGTGAGGGCATTGACCTTGAGTTCGAAGACTCTGGTCTGATGCCTGACACCAAGGACGTAATCCGCATCCTGCAAGCGCGTATCTATGAGCAATTAACGATTGACCTCTGGGAAGACGCAGAAGACTTGCTCAATGAATACTTG‑‑‑‑‑‑‑‑‑GAG‑‑‑‑‑‑‑‑‑GAAGTC‑‑‑‑‑‑‑‑‑Gcacgacagaaagaaattgaccgcgctaaggcccgtaaagaacgtcacgaggggcgcttagactgtctcttatacacatctccga                                                                                                                                                                                                            >  1:32807‑M1/1‑217 (MQ=255)`  
                                                                                              `aTTCACATGGCTGCCGATAATGCAGTTCCGCACTACTACGCTGACATCTTTAGCGTAATGGCAAGTGAGGGCATTGACCTTGAGTTCGAAGACTATGGTCTGATGCCTGACACCAAGGACGTAATCCGCATCCTGCAAGCGCGTATCTATGAGCAATTAACGATTGACCTCTGGGAAGACGCAGAAGACTTGCTCAATGAATACTGG‑‑‑‑‑‑‑‑‑GAG‑‑‑‑‑‑‑‑‑GAAGTCTCGGAAGTAGcacgacagaaagaaattgaccgcgctaaggcccgtaaagaacgtcccgaggggcgcttcgtggacagcagcgtca                                                                                                                                                                                                                     >  1:67593‑M1/1‑226 (MQ=255)`  
                                                                                                       `gCTGCCGATAATGCAGTTCCGCACTACTACGCTGACATCTTTAGCGTAATGGCAAGTGAGGGCATTGACCTTGAGTTCGAAGACTCTGGTCTGATGCCTGACACCAAGGACGTAATCCGCATCCTGCAAGCGCGTATCTATGAGCAATTAACGATTGACCTCTGGGAAGACGCAGAAGACTTGCTCAATGAATACTTGGAGGAAGTCGAG‑‑‑‑‑‑‑‑‑GAAGTC‑‑‑‑‑‑‑‑‑Gcacgacagaaataaattgaccgcgctaaggcccgtaaagaacgtcacgaggggcgcttagaggcacgcagattcaaacctgtcc                                                                                                                                                                                                            >  1:134685‑M1/1‑217 (MQ=255)`  
                                                                                                        `cTGCCGATAATGCAGTTCCGCACTACTACGCTGACATCTTTAGCGTAATGGCAAGTGAGGGCATTGACCTTGAGTTCGAAGACTCTGGTCTGATGCCTGACACCAAGGACGTAATCCGCATCATGCAAGCACGTATCTATGAGCAATTAACGATTGACCTCTGGGAAGACGCAGAAGACTTGCTCAATGAATACTTG‑‑‑‑‑‑‑‑‑GAG‑‑‑‑‑‑‑‑‑GAAGTC‑‑‑‑‑‑‑‑‑Gcacgacagaaagaaattgaccgcgctaaggcccgtaaagaacgtcacgaggggcgcttagaggcacgcagattcaaacgtcgcaaccgcaaggc                                                                                                                                                                                                  <  1:189693‑M1/301‑95 (MQ=255)`  
                                                                                                              `aTAATGCAGTTCCGCACTACTACGCTGACATCTTTAGCGTAATGGCAAGTGAGGGCATTGACCTTGAGTTCGAAGACTCTGGTCTGATGCCTAACACCAAGGACGAAATCCGCATCCTGCAAGCGCGTATCTATGAGCAATTAACGATTGACCTCTGGGAAGACGCAGAAGACTTGCTCAATGAATACTTT‑‑‑‑‑‑‑‑‑GAG‑‑‑‑‑‑‑‑‑GAAGTC‑‑‑‑‑‑‑‑‑Gcacgccagaaagaaattgaccgcgctaaggcccgtaaagaacgtcacgaggggcgcttagaggcacgcagattcaaacgtcgcaaccgcacggcacgaaa                                                                                                                                                                                            >  1:204181‑M1/1‑201 (MQ=255)`  
                                                                                                                `ggctcggagatgtgtataagagacagcatcgttagcgtaatggcaagtgagggCATTGACCTTGAGTTCGAAGACTCTGGTCTGATGCCTGACACCAAGGACGTAATCCGCATCCTGCAAGCGCGTATCTATGAGCAATTAACGATTGACCTCTGGGAAGACGCAGAAGACTTGCTCAATGAATACTTG‑‑‑‑‑‑‑‑‑GAG‑‑‑‑‑‑‑‑‑GAAGTC‑‑‑‑‑‑‑‑‑Gcacgacagaaagaaattgaccgcgctaaggcccgtaaagaacgtaacgaggggcgcttagaggcacgcagattcaaacgtcgcaaccgcaaggcacgtaaag                                                                                                                                                                                          <  1:271590‑M1/275‑103 (MQ=255)`  
                                                                                                                 `aTGCAGTTCCGCACTACTACGCTGACATCTTTAGCGTAATGGCAAGTGAGGGCATTGACCTTGAGTTCGAAGACTCTGGTCTGATGCCTGACACCAAGGACGTAATCCGCATCCTGCAAGCGCGTATCTATGAGCAATTAACGATTGACCTCTTGGAAGACGCAGAAGACTTGCTCAATGAATACTTGGAGGAAGTCGAG‑‑‑‑‑‑‑‑‑GAAGTC‑‑‑‑‑‑‑‑‑Gcacgacagaaagaaattgaccgcgctaaggcccgtaaagaacgtcccgaggggcgcttagaggcacgcagattcaaacgtcgcaaccgcaaggc                                                                                                                                                                                                  >  1:312817‑M1/1‑207 (MQ=255)`  
                                                                                                                 `aTGCAGTTCCGCACTACTACGCTGACATCTTTAGCGTAATGGCAAGTGAGGGCATTGACCTTGAGTTCGAAGACTCTGGTCTGATGCCTGACACCAAGGACGTAATCCGCATCCTGCAAGCGCGTATCTATGAGCAATTAACGATTGACCTCTGGGAAGACGCAGAAGACTTGCTCAATGAATACTTGGAGGAAGTCGAG‑‑‑‑‑‑‑‑‑GAAGTC‑‑‑‑‑‑‑‑‑Gcacgacagaaagaaattgaccgcgctaaggcccgtaaagaacgtcacgaggggcgcttagaggcacgcagattcaaacgtcgcaaccgcaaggc                                                                                                                                                                                                  >  1:213964‑M1/1‑207 (MQ=255)`  
                                                                                                                 `aTGCAGTTCCGCACTACTACGCTGACATCTTTAGCGTAATGGCAAGTGAGGGCATTGACCTTGAGTTCGAAGACTCTGGTCTGATGCCTGACACCAAGGACGTAATCCGCATCCTGCAAGCGCGTATCTATGAGCAATTAACGATTGACCTCTGGGAAGACGCAGAAGACTTGCTCAATGAATACTTG‑‑‑‑‑‑‑‑‑GAG‑‑‑‑‑‑‑‑‑GAAGTC‑‑‑‑‑‑‑‑‑Gcacgacagaaagaaattgaccgcgctaaggcccgtaaagaacgtcacgaggggcgcttagaggcccgcagattcaaacgtcgcaaccgcaaggcacgtaaagc                                                                                                                                                                                         >  1:252956‑M1/1‑198 (MQ=255)`  
                                                                                                                 `aTGCAGTTCCGCACTACTACGCTGACATCGTTAGCGTAATGGCAAGTGAGGGCATTGACCTTGAGTTCGAGGACTCTGGTCTGATGCCTGACACCAAGGCCGTAATCCGCATCCTGCAAGCGCGTATCTATGAGCAATTAACGATTGACCTCTGGGAAGACGCAGAAGACTTGCTCAATGAATACTTG‑‑‑‑‑‑‑‑‑GAG‑‑‑‑‑‑‑‑‑GAAGTC‑‑‑‑‑‑‑‑‑Gcacgacagaaagaaattgaccgcgctaaggcccgtaaagaacgtcacgaggggcgcttagaggcacgcagattcaaacgtcgcaaccgcaaggcacgtaaagc                                                                                                                                                                                         <  1:29072‑M1/301‑104 (MQ=255)`  
                                                                                                                       `ttCCGCACTACTACGCTGACATCTTTAGCGTAATGGCAAGTGAGGGCATTGACCTTGAGTTCGAAGACTCTGGTCTGATGCCTGACACCAAGGACGTAATCCGCATCCTGCAAGCGCGTATCTATGAGCAATTAACGATTGACCTCTGGGAAGACGCAGAAGACTTGCTCAATGAATACTTG‑‑‑‑‑‑‑‑‑GAG‑‑‑‑‑‑‑‑‑GAAGTC‑‑‑‑‑‑‑‑‑Gcacgacagaaagaaattgaccgcgctaaggcccgtaaagaacgtcatgaggggcgcttagatgcacgcagattcaaacgtcgcaaccgcaaggcacgtaaagcacacaa                                                                                                                                                                                   <  1:37110‑M1/301‑110 (MQ=255)`  
                                                                                                                         `ccGCACTACGCCGCTGACATCTTTAGCCTAATGGCAAGTGAGGGCATTGACCTTGAGTTCGAAGACTCTGGTCTGATGCCTGACACCAAGGACGTAATCCGCATCCTGCAAGCGCGTATCTATGAGCAATTAACGATTGACCTCTGGGAAGACGCAGAAGACTTGCTCAATGAATACTTG‑‑‑‑‑‑‑‑‑GAG‑‑‑‑‑‑‑‑‑GAAGTC‑‑‑‑‑‑‑‑‑Gaggaagtcgaacgacagaaagaaattgaccgcgctaaggcccgtaaagaacgtcacgaggggcgcttagaggcacgcagattcaaacgtcgcaaccgcaaggcacgtaaag                                                                                                                                                                                 <  1:184086‑M1/301‑112 (MQ=255)`  
                                                                                                                           `gatgtgtataagagacagctttagcgtAATGGCAAGTGAGGGCATTGACCTTGAGTTCGAAGACTCTGGTCTGATGCCTGACACCAAGGACGTAATCCGCATCCTGCAAGCGCGTATCTATGAGCAATTAACGATTGACCTCTGGGAAGACGCAGAAGACTTGTTCAATGAATACTTG‑‑‑‑‑‑‑‑‑AAG‑‑‑‑‑‑‑‑‑GAAGTC‑‑‑‑‑‑‑‑‑Gcacgacagaaagaaattgaccgcgctaaggcccgtaaagaacgtcacgaggggcgcttagaggcacgcagattcaaacgtcgcaaccgcaaggcacgtaaagcacacaaagct                                                                                                                                                                               <  1:144466‑M1/288‑114 (MQ=255)`  
                                                                                                                            `cACTACTACGCTGACATCTTTAGCGTAATGGCAAGTGAGGGCATTGACCTTGAGTTCGAAGACTCTGGTCTGATGCCTGACACCAAGGACGTAATCCGCATCCTGCAAGCGCGTATCTATGAGCAATTAACGATTGACCTCTGGGAAGACGCAGAAGACTTGCTCAATGAATACTTGGAGGAAGTCGAG‑‑‑‑‑‑‑‑‑GAAGTC‑‑‑‑‑‑‑‑‑Gcacgacagaaagaaattgaccgcgctaaggcccgtaaagaacgtcacgaggggcgcttagaggcacgcagattcaaacgtcgcaaccgcaaggcacgtaacgctg                                                                                                                                                                                       >  1:333321‑M1/1‑196 (MQ=255)`  
                                                                                                                              `ctactaCGCTGACATCTTTAGCGTAATGGCAAGTGAGGGCATTGACCTTGAGTTCGAAGACTCTGGTCTGATGCCTGACACCAAGGACGTACTCCGCATCCTGCAAGCGCGTATCTATGAGCAATTAACGATTGACCTCTGGGCAGACGCAGAAGACTTGCTCAATGAATACTTGGAGGAAGTCGAG‑‑‑‑‑‑‑‑‑GAAGTC‑‑‑‑‑‑‑‑‑Gcacgacatccagaaattgaccgcgctaaggcccgtaaagaacgtcccgaggggcgcttagaggcacgcagcttcaaacgtcgcaaccgcaaggcccgtaaagcacac                                                                                                                                                                                     >  1:56452‑M1/1‑194 (MQ=255)`  
                                                                                                                              `ctactaCGCTGACATCTTTAGCGTAATGGCAAGTGAGGGCATTGACCTTGAGTTCGAAGACTCTGGTCTGATGCCTGACACCAAGGACGTAATCCGCATCCTGCAAGCGCGTATCTCTGAGCAATTAACGATTGACCTCTGGGAAGACGCAGAAGACTTGCTCAATGAATCCTTG‑‑‑‑‑‑‑‑‑GAG‑‑‑‑‑‑‑‑‑GAAGTC‑‑‑‑‑‑‑‑‑Gcacgacagaaagaaattgaccgcgctaaggcccgtaaagcacgtcacgaggggcgcttagaggcacgcagattcaaacgtcgcaaccgccaggcccgtaaagcacactaagctaag                                                                                                                                                                            >  1:228009‑M1/1‑185 (MQ=255)`  
                                                                                                                              `ctactaCGCTGACATCTTTAGCGTAATGGCAAGTGAGGGCATTGACCTTGAGTTCGAAGACTCTGGTCTGATGCCTGACACCAAGGACGTAATCCGCATCCCGCAAGCGCGTATCTATGAGCAATTAACGATTGACCTCTGGGAAGACGCAGAAGACTTGCTCAATGAATACTTGGAGGAAGTCGAG‑‑‑‑‑‑‑‑‑GAAGTC‑‑‑‑‑‑‑‑‑Gcacgacagaaagaaattgaccgcgctaaggcccgtaacgaacgtcacgaggggcgcttagaggcacggagattcaaacgtcgcaaccgcaagcacgtaaagcacaca                                                                                                                                                                                     >  1:19972‑M1/1‑194 (MQ=255)`  
                                                                                                                                        `gACATCTTTAGCGTAATGGCAAGTGAGGGCATTGACCTTGAGTTCGAAGACTCTGGTCTGATGCCTGACACCAAGGACGTAATCCGCATCCTGCAAGCGCGTATCTATGAGCAATTAACGATTGACCTCTGGGAAGACGCAGAAGACTTGCTCAATGAATACTTG‑‑‑‑‑‑‑‑‑GAG‑‑‑‑‑‑‑‑‑GAAGTC‑‑‑‑‑‑‑‑‑Gcacgacagaaagaaattgaccgcgctaaggcccgtaaagaacgtcacgagtggcgcttagaggcacgcagattcaaacgtcgcaaccgcaaggcacgtaaagcacacaaagctaagcgcgaaagaa                                                                                                                                                                  >  1:181296‑M1/1‑175 (MQ=255)`  
                                                                                                                                                       `aTGGCAAGTGAGGGCATTGACCTTGAGTTCGAAGACTCTGGTCTGATGCCTGACACCAAGGACGTAATCCGCATCCTGCAAGCGCGTATCTATGAGCAATTGACGATTGACCTCTGGGAAGACGCAGAAGACTTGCTCAATGAATACTTGGAGGAAGTCGAG‑‑‑‑‑‑‑‑‑GAAGTC‑‑‑‑‑‑‑‑‑Gcacgacagaaagaaattgaccgcgctaaggcccgtaaagaacgtcacgaggggcgcttagaggcgcgcagattcaaacgtcgcaaccgcaaggcaagtaaagcacacaaagctaagagcaaaagaatgcttt                                                                                                                                                            >  1:41981‑M1/1‑169 (MQ=255)`  
                                                                                                                                                        `tGGCAAGTGAGGGCATTGACCTTTAGTTCGAAGACTCTGGTCTGATGCCTGACACCAAGGACGTAATCCGCATCCTGCAAGCGCGTATCTATGAGCAATTAACGATTGACCTCTGGGAAGACGCAGAAGACTTGCTCAATGAATACTTGGAGGAAGTCGAG‑‑‑‑‑‑‑‑‑GAAGTC‑‑‑‑‑‑‑‑‑Gcacgacagaaagaaattgaccgcgctaaggcccgtaaagaacgtcacgaggggcgcttagaggcacgcagattcaaacgtcgcaaccgcaaggcacgtaaagcacacaaagataagggcgaaagaatgcttgc                                                                                                                                                           >  1:156541‑M1/1‑168 (MQ=255)`  
                                                                                                                                                        `tGGCAAGTGAGGGCATTGACCTTGAGTTCGAAGACTCTGGTCTGATGCCTGACACCAAGGACGTAATACGCATCCTGCAAGCGCGTATCTATGAGCAATTAACGATTGACCTCTGGGAAGACGCAGAAGACTTGCTCAATGAATACTTG‑‑‑‑‑‑‑‑‑GAG‑‑‑‑‑‑‑‑‑GAAGTC‑‑‑‑‑‑‑‑‑Gcacgacagaaagaaattgaccgcgctaaggcccgtaaagaacgtcacgaggggcgcttagaggcacgcagattcaaacgtcgcaaccgcaaggtacgtaaagcacacaaagctaagcgcgaaagaatgcttgctgcgtggcg                                                                                                                                                  >  1:131238‑M1/1‑159 (MQ=255)`  
                                                                                                                                                              `gTGAGGGCATTGACCTTGAGTTCGAAGACTCTGGTCTGATGCCTGACACCAAGGACGTAATCCGCATCCTGCAAGCGCGTATCTATGAGCAATTAACGATTGACCTCTGGGAAGACGCAGATGACTTGCTCAATGAATACTTG‑‑‑‑‑‑‑‑‑GAG‑‑‑‑‑‑‑‑‑GAAGTC‑‑‑‑‑‑‑‑‑Gcacgacagaaagaaattgaccgcgctaaggcccgtaaagaacgtcacgaggggcgcttagaggcacgcagattcaaacgtcgcaacagcaaggcacgtaaagcacacaaagctaagcgcgaaagaatgcttgctgcgtggcgatgggc                                                                                                                                            <  1:303816‑M1/301‑149 (MQ=255)`  
                                                                                                                                                                   `tataagagacaggagttcgaatactCTGGTCTGATGCCTGACACCAAGGACGTAATCCGCATCCTGCAAGCGCGTATCTATGAGCAATTAACGATTGACCTCTGGGAAGACGCAGAAGACTTGCTCAATGAATACTTG‑‑‑‑‑‑‑‑‑GAG‑‑‑‑‑‑‑‑‑GAAGTC‑‑‑‑‑‑‑‑‑Gcacgacagaaagaaattgaccgcgctaaggcccgtaaagaacgtcacgagaggcgcttagaggcacgcagattcaaacgtcgcaaccgcaaggcacgtaaagcacacaaagctaagcgcgaaagaatgcttgctgcgtggcgatgggctgaac                                                                                                                                       <  1:75860‑M1/289‑154 (MQ=255)`  
                                                                                                                                                                            `cTTGAGTTCGAAGACTCTGGTCTGATGCCTGACACCAAGGACGTAATCCGCATCCTGCAAGCGCGTATCTATGAGCAATTAACGATTGACCTCTGGGAAGACGCAGAAGACTTGCTCAATGAATACTTG‑‑‑‑‑‑‑‑‑GAG‑‑‑‑‑‑‑‑‑GAAGTC‑‑‑‑‑‑‑‑‑Gcacgacagaaagaaattgaccgcgctaaggcccgtaaagaacgtcacgaggggcgcttagaggcacgcagattcaaacgtcgcaaccgcaaggcacgtaaagcacacaaagctaagcgcgaaagaatgcttgctgcgtggcgatgggctgaacgtcaagaac                                                                                                                              <  1:321748‑M1/301‑163 (MQ=255)`  
                                                                                                                                                                                    `cGAAGACTTTGGTCTGATGCCTGACACCAAGGACGTAATCCGCATCCTGCAAGCGCGTATCTATGAGCAATTAACGATTGACCTCTGGGAAGACGCAGAAGACTTGCTCAATGAATACTTGGAGGAAGTCGAG‑‑‑‑‑‑‑‑‑GAAGTC‑‑‑‑‑‑‑‑‑Gcacgacagaaagaaattgaccgcgctaaggcccgtaaagaacgtcacgaggggcgcttagaggcacgcagattcaaacgtcgcaaccgcaaggcacgtaaagcacacaaagctaagcgcgaaagaatgcttgctgcgtggcgatgggctgaacgtcaagaa                                                                                                                               <  1:187017‑M1/301‑162 (MQ=255)`  
                                                                                                                                                                                      `aaGACTCTGGGCTGATGCCTGAAAACAAGGACGTAATCCGAATCCTGCAAGAGCGTATCTATGAGCAATTAACGATTGACCTCTGGGAAGACGCAGAAGACTTGCTCAATGAATACTTG‑‑‑‑‑‑‑‑‑GAG‑‑‑‑‑‑‑‑‑GAAGTC‑‑‑‑‑‑‑‑‑Gcacgacagaaagaaattgaccgcgctaaggcccgtaaagaacgtcacgaggggcgcttagaggcacgcagattcaaacgtcgcaaccgcaaggcacgtaaagcacacaaagctaagcgcgaaagaatgcttgctgcgtggcgatgggctgaacgtcaagaacggcgtaacca                                                                                                                    <  1:92694‑M1/301‑173 (MQ=255)`  
                                                                                                                                                                                         `aCTCTGGTCTGATGCCTGACACCAAGGACGTAATCCGCATCCTGCAAGCGCGTATCTATGAGCAATTAACGATTGACCTCTGGGAAGACGCAGAAGACTTGCTCAATGAATACTTGGAGGAAGTCGAG‑‑‑‑‑‑‑‑‑GAAGTC‑‑‑‑‑‑‑‑‑Gcacgacagaaagaaattgaccgcgctaaggcccgtaaagaacgtcacgaggggcgcttagaggcacgcagattcaaacgtcgcaaccgcaaggcacgtaaagcacacaaagctaagcgcgaaagaatgcttgctgcgtggcgatgggctgaacgtcaagaacggcg                                                                                                                          >  1:50830‑M1/1‑135 (MQ=255)`  
                                                                                                                                                                                               `aaggagaagtgtagaagtgacaggtaatcctcttcctgcaagcgcgtATCTATGAGTAATTAACGATTTACCTCTGGGAAGACGCAGAAAACTTGCACAATGAATACTTGGGGGAAGTCGAG‑‑‑‑‑‑‑‑‑GAAGTC‑‑‑‑‑‑‑‑‑Gcacgacagaaagaaattgaccgcgctaaggcccgtaaagaacgtcacgaggggcgcttataggcacgcagattcaaacgtcgcaaccgcaaggcacgtaaagcacacaaagctaagcgcgaaagaatgcttgctgcgtggcgatgggctgaacgtcaagaacggcgtaacca                                                                                                                    <  1:4503‑M1/278‑173 (MQ=255)`  
                                                                                                                                                                                                   `gATGCCTGACACCAAGGACGTAATCCGCATCCTGCAAGCGCGTATCTATGAGCAATTAACGATTGACCTCTGGGAAGACGCAGAAGACTTGCTCAATGAATACTTG‑‑‑‑‑‑‑‑‑GAG‑‑‑‑‑‑‑‑‑GAAGTC‑‑‑‑‑‑‑‑‑Gcacgacagaaagaaattgaccgcgctaaggcccgtaaagaacgtcactaggggcgcttagaggcacgcagattcaaacgtcgcaaccgcaaggcacgtaaagcacacaaagctaagcgcgaaagaatgcttgctgcgtggcgatgggcttaacgtcaagaacggcgtaaccatgaggtagctgta                                                                                                       >  1:33992‑M1/1‑116 (MQ=255)`  
                                                                                                                                                                                                      `gCCTGACACCAAGGACGTAATCCGCATCCTGCAAGCGCGTATCTATGAGCAATTAACGATTGACCTCTGGGAAGACGCAGAAGACTTGCTCAATGAATACTTG‑‑‑‑‑‑‑‑‑GAG‑‑‑‑‑‑‑‑‑GAAGTC‑‑‑‑‑‑‑‑‑Gcacgacagaaagaaattgaccgcgctaaggcccgtaaagaacgtcacgaggggcgcttagaggcacgcagattcaaacgtcgcaaccgcaaggcacgtaaagcacacaaagctaagcgcgaaagaatgcttgctgcgtggcgatgggctgaacgtcaagaacggcgtaaccatgaggtagctgtagat                                                                                                    <  1:232891‑M1/301‑189 (MQ=255)`  
                                                                                                                                                                                                      `gCCTGACACCAAGGACGTAATCCGCATCCTGCAAGCGCGTATCTATGAGCAATTAACGATTGACCTCTGGGAAGACGCAGAAGACTTGCTCAATGAATACTTG‑‑‑‑‑‑‑‑‑GAG‑‑‑‑‑‑‑‑‑GAAGTC‑‑‑‑‑‑‑‑‑Gcacgacagaaagaaattgaccgcgctaaggcccgtaaagaacgtcacgaggggcgcttagaggcacgcagattcaaacgtcgcaaccgcaaggcacgtaaagcacacaaagctaagcgcgaaagaatgcttgctgcgtggcaatgggctgaacgtcaagaactgtctcttatacacatctccgagccc                                                                                                    >  1:124387‑M1/1‑113 (MQ=255)`  
                                                                                                                                                                                                       `gacagcaccaaggACGTAATCCGCATACTGCAAGCGCGTATCTATGAGCAATTAACGATGGACCTCTGGGAAGACGCAGAAGACTTGCTCAATGAATACTTG‑‑‑‑‑‑‑‑‑GAG‑‑‑‑‑‑‑‑‑GAAGTC‑‑‑‑‑‑‑‑‑Gcacgacagaaagaaattgaccgcgctaaggcccgtaaagaacgtcacgaggggcgcttagaggcacgcagattcaaacgtcgcaaccgcaaggcacgtaaagcacacaaagctaagcgcgaaagaatgcttgctgcgtggcgatgggctgaacgtcaagaacggcgtaaccatgaggtagctgtagatg                                                                                                   <  1:306649‑M1/296‑190 (MQ=255)`  
                                                                                                                                                                                                       `ccTGACACCAAGGACGTAATCCGCATCCTGCAAGCGCGTATCTATGAGCAATTAACGATTGACCTCTTGGAAGACGCAGAAGACTTGCTCAATGAATACTTG‑‑‑‑‑‑‑‑‑GAG‑‑‑‑‑‑‑‑‑GAAGTC‑‑‑‑‑‑‑‑‑Gcacgacagaaagaaattgaccgcgctaaggcccgtaaagaacgtcacgaggggcgcttagaggcacgcagattcaaacgtcgcaaccgcaaggcacgtaaagcacacaaagctaagcgtgaaagaatgcttgctgcgtggcgatgggctgaacgtcaagaacggcgtaaccatgaggtagctgtagatg                                                                                                   <  1:127456‑M1/301‑190 (MQ=255)`  
                                                                                                                                                                                                       `ccTGACACCAAGGACGTAATCCGCATCCTGCAAGCGCGTATCTATGAGCAATTAACGATTGACCTCTGGGAAGACGCAGAAGACTTGCTCAATGAATACTTG‑‑‑‑‑‑‑‑‑GAG‑‑‑‑‑‑‑‑‑GAAGTC‑‑‑‑‑‑‑‑‑Gcacgacagaaagaaattgaccgcgctaaggcccgtaaagaacgtcacgaggggagcttagaggcacgcagattcaaacgtcgcaaccgcaaggcacgtaaagcacacaaagctaagcgcgaaagaatgcttgctgcgtggcgatgggctgaacgtcaagaacggcgtaaccatgaggtagatgtagatg                                                                                                   <  1:276976‑M1/301‑190 (MQ=255)`  
                                                                                                                                                                                                            `cacCAAGGACGTAATCCGCATCCTGCAAGCGCGTATCTATGAGCAATTAACGATTGACCTCTGGGAAGACGCAGAAGACTTGCTCAATGAATACTTG‑‑‑‑‑‑‑‑‑GAG‑‑‑‑‑‑‑‑‑GAAGTC‑‑‑‑‑‑‑‑‑Gcacgacagaaagaaattgaccgcgctaaggcccgtaaagaacgtcacgaggggcgcttagaggcacgcagattcaaacgtcgcaaccgcaaggcacgtaaagcacacaaagctaagcgcgaaagaatgcttgctgcgtggcgatgggctgaacgtcaagaacggcgtaaccatgaggtagctgtagatgtacta                                                                                              >  1:109492‑M1/1‑107 (MQ=255)`  
                                                                                                                                                                                                                `aaGGACGTAATCCGCATCCTGCAAGCGCGTATCTATGAGCAATTAACGATTGACCTCTGGGAAGACGAAGAAGACTTGCTCAATGAATACTTGGAGGAAGTCGAG‑‑‑‑‑‑‑‑‑GAAGTC‑‑‑‑‑‑‑‑‑Gcacgacagaaagaaattgaccgcgctaaggcccgtaaagaacgtcacgaggggcgcttagaggcacgcagattcaaacgtcgcaaccgcaaggcacgtaaagcacacaaagctaagcgcgaaagaatgcttgctgcgtggcgatgggctgaacgtcaagaacggcgtaaccatgaggtagctgtagatg                                                                                                   <  1:250445‑M1/301‑190 (MQ=255)`  
                                                                                                                                                                                                                  `ggACGTAATCCGCATCCTGCAAGCGCGTATCTATGAGCAATTAACGATTGACCTCTGGGAAGACGCAGAAGACTTGCTCAATGAATACTTG‑‑‑‑‑‑‑‑‑GAG‑‑‑‑‑‑‑‑‑GAAGTC‑‑‑‑‑‑‑‑‑Gcacgacagaaagaaattgaccgcgctaaggcccgtaaagaacgtcacgaggggcgcttagaggcacgcagattcaaacgtcgcaaccgcaaggcacgtaaagcacacaaagctaagcgcgaaagaatgcttgctgcgtggcgatgggctgaacgtcaagaacggcgtaaccatgaggtagctgtagatgtactaggaaga                                                                                        >  1:164736‑M1/1‑101 (MQ=255)`  
                                                                                                                                                                                                                     `cGTAATCCGCATCCTGCAAGCGCGTATCTATGAGCAATTAACGATTGACCTCTGGGAAGATGCAGAAGACTTGTTCAATGAATAATTG‑‑‑‑‑‑‑‑‑CAG‑‑‑‑‑‑‑‑‑GAAGTC‑‑‑‑‑‑‑‑‑Gcacgacagaaagaaattgaccgcgctaaggcccgtaaagaacgtcacgagggccgattagcggcacgcagattcaaacgtcgcaaccgcaagccacgtaaagcacacaaagctaagcgcgaaagaatgcgtgcttcgtggcgatgggccgaacgtcaagaacggcgtaacaaagaggtagctggatatttacctttctctttt                                                                                     >  1:147575‑M1/1‑98 (MQ=255)`  
                                                                                                                                                                                                                        `tcggagatttgtataagagacagtctatgagcaattaacgattgccaTCTGGGAAGACGCAGAAGACTTGCTCAATGAATACTTGGAGGAAGTCGAG‑‑‑‑‑‑‑‑‑GAAGTC‑‑‑‑‑‑‑‑‑Gcacgacagaaagaaattgaccgcgctaaggcccgtaaagaacgtcacgaggggcgcttagaggcacgcagattcaaacgtcgcaaccgcaaggcacgtaaagcacacaaagctaagcgcgaaagaatgcttgctgcgtggcgatgggatgaacgtcaagaacggcgtaaccatgaggtagctgtagatgtactagga                                                                                           <  1:315452‑M1/278‑198 (MQ=255)`  
                                                                                                                                                                                                                        `attccGCATCCGTCAAGCGCGTATTCATTAGCAATTAACGATTGACCGCTGGGAAGACGAAGAAGACTTGCTCAATGAATACTTGGAGGAAGTCGAG‑‑‑‑‑‑‑‑‑GAAGTC‑‑‑‑‑‑‑‑‑Gcacgacagaaagaaattgaccccgctaaggaccgtaaagaacgtcacgaggggcgcttagaggcacgcagattcaaacgtcgcaaccgcaaggcacgtaaagcacacaaagctaagcgcgaaagaatgcttgctgcgtggcgatgggctgaacgtcaagaacggcgtaaccatgaggtagctgtagatgtactagga                                                                                           <  1:178715‑M1/299‑198 (MQ=255)`  
                                                                                                                                                                                                                        `aataggcatccTGCAAGCGCGTATCTATGAGCAATTAACGATTGACCTCTGGGAAGACGCAGAAGACTTGCTCAATGAATACTTGGAGGAAGTCGAG‑‑‑‑‑‑‑‑‑GAAGTC‑‑‑‑‑‑‑‑‑Gcacgacagaaagaaattgaccgcgctaaggcccgtaaagaacgtcacgaggggcgcttagaggcacgcagattcaaacgtcgcaaccgcaaggcacgtaaagcacacaaagctaagcgcgaaagaatgcttgctgcgtggcgatgggctgaacgtcaagaacggcgtaaccatgaggtagctgtagatgtactagga                                                                                           <  1:112650‑M1/296‑198 (MQ=255)`  
                                                                                                                                                                                                                        `aaTCCGCATCCTGCAAGCGCGTATCTATGAGCAATTAACGATTGACCTCTGGGAAGACGCAGAAGACTTGCTCAATGAATACTTGTATGAAGTCGAG‑‑‑‑‑‑‑‑‑GAAGTC‑‑‑‑‑‑‑‑‑Gcacgacagaaagaaattgaccgcgctaaggcccgtaaagaacgtcacgaggggcgcttagaggcacgcagaatcaaacgtcgcaaccgcaaggcacgtaaagcacacaaagctaagcgcgaaagaatgcttgctgcgtggcgatgggctgaacgtcaagaacggcgtaaccatgaggtagctgtagatgtactagga                                                                                           <  1:222229‑M1/301‑198 (MQ=255)`  
                                                                                                                                                                                                                        `aaTCCGCATCCTGCAAGCGCGTATCTATGAGCAATTAACGATTGACCTCTGGGAAGACGCAGAAGACTTGCTCAATGAATACTTG‑‑‑‑‑‑‑‑‑GAG‑‑‑‑‑‑‑‑‑GAAGTC‑‑‑‑‑‑‑‑‑Gcacgacagaaagaaattgaccgcgctaaggcccgtaaagaacgtcacgaggggcgcttagaggcacgcagattcaaacgtagcaaccgcaaggcacgtaaagcacacaaagctaagcgcgaaagaatgcttgctgcgtggcgatgggctgaacgtcaagaacggcgtaaccatgaggtagctgtcgatgctgtccatcatacacaa                                                                                  >  1:309626‑M1/1‑95 (MQ=255)`  
                                                                                                                                                                                                                         `aTCCGCATCCTGCAAGCGCGTATCTATGAGCAATTAACGATTTACTTCTGGGAAGACGCAGAAGACTTGCTCAATGAATACTTGGAGGAAGTCGAG‑‑‑‑‑‑‑‑‑GAAGTC‑‑‑‑‑‑‑‑‑Gcacgacagaaagaaattgaccgcgctaaggcccgtaaagaacgtcacgaggggcgcttagaggcacgcagattcaaacgtagcaaccgcaaggcacgtaaagcacccaaagctaagcgcgaaagaatgcttgctgcgtggcgatgggctgaacgtcaagaacggcgtaaccaggaggtagctgtagatggactaggaa                                                                                          >  1:149943‑M1/1‑103 (MQ=255)`  
                                                                                                                                                                                                                          `cccgCATCCTGCAAGCGCGTATCTATGAGCAATTAACGATTGACCTCTGGGAAGACGCAGAAGACTTGCTCAATGAATACTTG‑‑‑‑‑‑‑‑‑GAG‑‑‑‑‑‑‑‑‑GAAGTA‑‑‑‑‑‑‑‑‑Gcacgacagaaagaaattgaccgcgctaaggcccgtaaagaacgtcacgaggggcgcttagaggcacgcagattcaaacgtcgcaaccgcaaggcacgtaaagcacacaaagctaagcgcgaaagaatgcttgctgcgtggcgatgggctgaacgtcaagaacggcgtaaccatgaggtagctgtagatgtactaggaagaaccaataa                                                                                <  1:287803‑M1/300‑209 (MQ=255)`  
                                                                                                                                                                                                                             `gCAACCTGCAAGCGCGTATCTACGAGCAATTAACGATTGACCTCTAGGAAAACGCAGAAGACTTGCTCAATGAAAGCTTG‑‑‑‑‑‑‑‑‑GAG‑‑‑‑‑‑‑‑‑GAAGTA‑‑‑‑‑‑‑‑‑Gcacgacagaaaaaaattgaccgcgctaaggcccgtaaagaaagtcacgaggtgcgcttagaggcacgcagattcaaacgtcgcaaccgcaaggaacgtaaagcacacaaagctaagcgcgaaagaatgcttgctgcgtggcgatgggctgaacgtcaagaacggcgtaaccatgaggtagctgtagatgtactaggaagaaccaataacgc                                                                             <  1:29788‑M1/301‑212 (MQ=255)`  
                                                                                                                                                                                                                                `tCCTGCAAGCGCGTATCTATGAGCAATTAACGATTGACCTCTGGGAAGACGCAGAAGACTTGCTCAATGAATACTTGGAGGAAGTCGAG‑‑‑‑‑‑‑‑‑GAAGTC‑‑‑‑‑‑‑‑‑Gcacgacagaaagaaattgaccgcgctaaggcccgtaaagaacgtcacgaggggcgcttagaggcacgcagattcaaacgtcgcaaccgcaaggtacgtaaagcacacaaagctaagcgcgaaagaatgcttgctgcgtggcgattggctgaacgtcaagaacggcgtaaccatgaggtagctgtagatgtacctgtctcttatac                                                                                   >  1:245308‑M1/1‑96 (MQ=255)`  
                                                                                                                                                                                                                                  `cTGCAAGCGCGTATCTATGAGCAATTAACGATTGACCTCTGGGAAGACGCAGAAGACTTGCTCAATGAATACTTGGAGGAAGTCGAG‑‑‑‑‑‑‑‑‑GAAGTC‑‑‑‑‑‑‑‑‑Gcacgacagaaagaaattgaccgcgctaaggcccgtaaagaacgtcacgaggggctcttagaggcacgcagattcaaacgtcgcaaccgcaaggcacgtaaagcacacaaagctaagcgcgaaagaatgcttgctgcgtggctatgggctgaacgtcaagaacggcgtaaccatgaggtagctgtagatctgtctcttatacacatct                                                                                 >  1:106341‑M1/1‑94 (MQ=255)`  
                                                                                                                                                                                                                                  `cTGCAAGCGCGTATCTATGAGCAATTAACGATTGACCTCTGGGAAGACGCAGAAGACTTGCTCAATGAATACTTG‑‑‑‑‑‑‑‑‑GAG‑‑‑‑‑‑‑‑‑GAAGTC‑‑‑‑‑‑‑‑‑Gcacgacagaaagaaattgaccgcgctaaggcccgtaaagaacgtcacgaggggcgcttagaggcacgcagattcaaacgtcgcaaccgcaaggcacgtaaagcacacaaagctaagcgcgaaagactgcttgctgcgtggctatgggctgaacgtcaagaacggcgtaaccatgaggtagctgtagatgtactaggactgtctcttatacacctct                                                                        >  1:283170‑M1/1‑85 (MQ=255)`  
                                                                                                                                                                                                                                  `cTGCAAGCGCGTATCTATGAGCAATTAACGATTGACCTCTGGGAAGACGCAGAAGACTTGCTCAATGAATACTTG‑‑‑‑‑‑‑‑‑GAG‑‑‑‑‑‑‑‑‑GAAGTC‑‑‑‑‑‑‑‑‑Gcacgacagaaagaaattgaccgcgctaaggcccgtaaagaacgtcacgaggggcgcttagaggcacgcagattcaaacgtcgcaaccgcaaggcacgtaaagcacacaaagctaagcgcgaaagaatgcttgctgcgtggcgatgggctgaacgtcaagaacggcgtaaccatgaggtagctgtagatgtactaggaagaaccaataacgctactt                                                                        >  1:175930‑M1/1‑85 (MQ=255)`  
                                                                                                                                                                                                                                  `cTGCAAGCGCGTATCTATGAGCAATTAACGATTGACCTCTGGGAAGACGCAGAAGACTTGCTCAATGAATACTTG‑‑‑‑‑‑‑‑‑GAG‑‑‑‑‑‑‑‑‑GAAGTC‑‑‑‑‑‑‑‑‑Gcacgacagaaagaaattgaccgagctaaggcccgtaaagaacgtcacgaggggcgcttagaggcacgcagattaaaacgtcgcaaccgcaaggcacgtaaagcacacaaagctaagcgcgaaagaatgcttgctgcgtggcgatgggctgaacgtcaagaacggcgtaaccatgaggtagctgtagatggactaggaagaaccaataacggtccct                                                                        >  1:115603‑M1/1‑85 (MQ=255)`  
                                                                                                                                                                                                                                  `atgCAAGCGCGTATCTATGAGCAATTAACGATTGACCTCTGGGAAGACGCAGAAGACTTGCTCAATTAATACTTG‑‑‑‑‑‑‑‑‑GAG‑‑‑‑‑‑‑‑‑GAAGTC‑‑‑‑‑‑‑‑‑Gcacgacagaaagaaattgaccgcgctaaggcccgtaaagaacgtcaagaggggcgcttagaggcacgcagattcaaacgtcgcaaccgcaaggcacgtaaagcacacaaagctaagcgcgaaagaatgcttgctgcgtggcgatgggcggaaagtcaagaacggcgtaaccatgaggtagcggtagatgtactaggaggaaccaataacggcggtc                                                                        >  1:262‑M1/2‑85 (MQ=255)`  
                                                                                                                                                                                                                                   `ggcAAGCGCGTATCTAGGAGCAATTAACGATTGACCTCTGGGAAGACGCAGAAGACTTGCTCAATGAATACTTGGAGGAAGTCGAG‑‑‑‑‑‑‑‑‑GAAGTC‑‑‑‑‑‑‑‑‑Gcacgacagaaagaaattgaccgcgctaaggcccgtaaagaacgtcacgaggggcgcttagaggcacgcagattcaaacgtcgcaaccgcaaggcacgtaaagcacacaaagctaagcgcgaaagaatgcttgctgcgtggcgatgggctgaacgtcaagaacggcgtaaccatgaggtagctgtagatgtactaggaagaaccaataa                                                                                <  1:268818‑M1/300‑209 (MQ=255)`  
                                                                                                                                                                                                                                       `aGCGCGTATCTATGAGCAATTAACGATTGACCTCTGGGAAGACGCAGAAGACTTGCTCAATGAATACTTGGAGGAAGTCGAG‑‑‑‑‑‑‑‑‑GAAGTC‑‑‑‑‑‑‑‑‑Gcacgacagaaagaaattgaccgcgctaaggcccgtaaagaacgtcacgaggggcgcttagaggcacgcagattcaaacgtcgcaaccgcaaggcacgtaaagcacacaaagctaagcgcgaaagaatgcttgctgcgtggcgatgggctgaacgtcaagaacggcgtaaccatgaggtagctggagatgtactaggaagaaccaataacgct                                                                            >  1:89847‑M1/1‑89 (MQ=255)`  
                                                                                                                                                                                                                                       `aGCGCGTATCTATGAGCAATTAACGATTGACCTCTGGGAAGACGCAGAAGACTTGCTCAATGAATACTTG‑‑‑‑‑‑‑‑‑GAGGAAGTCGATGAAGTC‑‑‑‑‑‑‑‑‑Gcacgacagaaagaaattgaccgcgctaaggcccgtaaagaacgtcacgaggggcgcttagaggcacgcagattcaaacgtcgcaaccgcaaggcacgtaaagcacacaaagctaagcgcgaaagaatgcttgctgcgtggcgatgggctgaacgtcaagaacggcgtaaccatgaggtagctgtagatgtactaggaagaaccaataacgct                                                                            >  1:278890‑M1/1‑89 (MQ=255)`  
                                                                                                                                                                                                                                       `aGCGCGTATCTATGAGCAATTAACGATTGACCTCTGGGAAGACGCAGAAGACTTGCTCAATGAATACTTG‑‑‑‑‑‑‑‑‑GAG‑‑‑‑‑‑‑‑‑GAAGTC‑‑‑‑‑‑‑‑‑Gcacgacagaaagaaattgaccgcgctaaggcccgtaaagaacgtcacgaggggcgcttagaggcacgcagattcaaacgtcgcaaccgcaaggcacgtaaagcacacaaagctaagcgcgaaagaatgcttgctgcgtggcgatgggctgaacgtcaagaacggcgtaaccatgaggtagctgtagatgtactaggaagaaccaataacgctatgctctgg                                                                   <  1:219296‑M1/301‑222 (MQ=255)`  
                                                                                                                                                                                                                                            `gTATCTATGAGCAATTAACGATTGACCTCTGGGAAGACGCAGAAGACTTGCTCAATGAATACTTGGAGGAAGTCGAG‑‑‑‑‑‑‑‑‑GAAGTC‑‑‑‑‑‑‑‑‑Gcacgacagaaagaaattgaccgcgctaaggcccgtaaagaacgtcacgaggggcgcttagaggcacgcagattcaaacgtcgcaaccgcaaggcacgtaaagcacacaaagctaagcgcgaaagaatgcttgctgcgtggcgatgggctgaacgtcaagaacggcgtaaccatgaggtagctgtagatgtactaggaagaaccaataacgcctgtcc                                                                       >  1:301523‑M1/1‑84 (MQ=255)`  
                                                                                                                                                                                                                                               `tCTATGAGCAATTAACGATTGACCTCTGGGAATACGCAGAAGACTTGCTCAATGAATACTTG‑‑‑‑‑‑‑‑‑GAG‑‑‑‑‑‑‑‑‑GAAGTC‑‑‑‑‑‑‑‑‑Gcacgacagaaagaaattgaccgcgctaagtcccgtaaagaatgtcacgaggggcgcttagaggcacgcagattcaaacgtcgcaaccgcaaggcacgtaaagcacacaaagctaagcgcgaaagaatgcttgctgcgtggcgatgggctgaacgtcaagaacggcgtaaccatgaggtagctgtagatgtactaggaagaaccaataacgctatgctcttggtcaacat                                                           >  1:274344‑M1/1‑72 (MQ=255)`  
                                                                                                                                                                                                                                               `tCTATGAGCAATTAACGATTGACCTCTGGGAAGACGCAGAAGACTTGCTCAATGAATACTTG‑‑‑‑‑‑‑‑‑GAT‑‑‑‑‑‑‑‑‑GAAGTC‑‑‑‑‑‑‑‑‑Gcacgacagaaagaaattgaccgcgctaaggcccgtaaagaacgtcacgaggggcgcttagaggcacgcagattcaaacgtcgcaaccgcaaggcacgtaaagcacacaaagctaagcgcgaaagaatgcttgctgcgtgtcgatgggctgaacgtcaagaacggcgtaaccatgatgtagctgtagatgtactaggaagaaccaataacgctatgccctgggtccacac                                                           >  1:172866‑M1/1‑72 (MQ=255)`  
                                                                                                                                                                                                                                                 `tATGAGCAATTAACGATTGACCTCTGGGAAGACGCAGAAGACTTGCTCAATGAATACTTG‑‑‑‑‑‑‑‑‑GAG‑‑‑‑‑‑‑‑‑GAAGTC‑‑‑‑‑‑‑‑‑Gcacgacagaaagaaattgaccgcgctaaggcccgtaaagaacgtcacgagtggcgcttagaggcacgcagattcaaacgtcgcaaccgcaaggcacgtaaagcacacaaagctaagcgcgaaagaatgcttgctgcgtggcgatgggctgaacgtcaagaacggcgtaaccatgaggtagctgtagatgtactaggaagaaccaataacgctatgctctgggtcaacatgt                                                         <  1:728‑M1/301‑232 (MQ=255)`  
                                                                                                                                                                                                                                                 `gagatgtgtataagagacagcctctgggaagacgcagaagacTTGCTCAATGAATACTTG‑‑‑‑‑‑‑‑‑GAG‑‑‑‑‑‑‑‑‑GAAGTC‑‑‑‑‑‑‑‑‑Gcacgacagaaagaaattgaccgcgctaaggcccgtaaagaacgtcacgaggggcgcttagaggcacgcagattcaaacgtcgcaaccgcaaggcacgtaaagcacacaaagctaagcgcgaaagaatgcttgctgcgtggcgatgggctgaacgtcaagaacggcgtaaccatgaggtagctgtagatgtactaggaagaaccaataacgctatgctctgggtcaacatgt                                                         <  1:235820‑M1/281‑232 (MQ=255)`  
                                                                                                                                                                                                                                                  `aTGAGCAATTAATGATTGACCGCTGGGAAGACGCAGAAGACTTGCTCAATGAATACTTGGAGGAAGGCGAG‑‑‑‑‑‑‑‑‑GAAGTC‑‑‑‑‑‑‑‑‑Gcacgacagaaagaaattgaccgcgctaaggcccgtaaagaacgtcacgaggggcgcttagaggcacgcagattcaaacgtcacaaccgcaaggcacgtaaagcacacaaagctaagcgcgaaagaatgcttgctgcgtggcgatgggctgaacgtcaagaacggcgtaaccatgaggtagctgtagatgtactaggaagaaccaataacgctatgctctgggt                                                                 <  1:121979‑M1/301‑224 (MQ=255)`  
                                                                                                                                                                                                                                                     `cgaaattaACGATTTACTTCTGGGAAGACGCAGAAGACTTGCTCAATGAATACTTG‑‑‑‑‑‑‑‑‑GAG‑‑‑‑‑‑‑‑‑GAAGTC‑‑‑‑‑‑‑‑‑Gcacgacagaaagatattgaccgcgctaaggcccgtaaagaacgtcacgaggggcgcttagaggcacgcagattcaaacgtcgcaaccgcaaggcacgtaaagcacacaaagctaagcgcgaaagaatgcttgctgcgtggcgatgggctgaacgtcaataactgcgtaaccatgaggtagctgtagatgtactaggaagaaccaataacgctatgctctgggtcaacatgttctc                                                     <  1:100236‑M1/298‑236 (MQ=255)`  
                                                                                                                                                                                                                                                     `aaagagacaggattgacctctGGGAAGACGCAGAAGACTTGCTCAATGAATACTTG‑‑‑‑‑‑‑‑‑GAG‑‑‑‑‑‑‑‑‑GAAGTC‑‑‑‑‑‑‑‑‑Gcacgacagaaagaaattgaccgcgctaaggcccgtaaagaacgtcacgaggggcgcttagaggcacgcagattcaaacgtcgcaaccgcaaggcacgtaaagcacacaaagctaagcgcgaaagaatgcttgctgcgtggcgatgggctgaacgtcaagaacggcgtaaccatgaggtagctgtagatgtactaggaagaaccaataacgctatgctctgggtcaacatgttctc                                                     <  1:336979‑M1/291‑236 (MQ=255)`  
                                                                                                                                                                                                                                                     `aGCAATTAAGGATTGACATCTGGGAAGACGCAGAAGCCTTGCTCAATGAATACTTG‑‑‑‑‑‑‑‑‑GAG‑‑‑‑‑‑‑‑‑GAAGTC‑‑‑‑‑‑‑‑‑Gcacgacagaaagaaattgaccgcgctaaggcccgtaaagaacgtcacgaggggcgcttagaggcacgcagattcaaacggcgcaaccgcaaggcacgtaaagcacacaaagctaagcgcgaaagaatgcttgctgcgtggcgatgggctgaacgtcaagaacggcgtaaccatgaggtagctgtagatgtactaggaagaaccaataacgctatgctctgggtcaacatgttctc                                                     <  1:7848‑M1/301‑236 (MQ=255)`  
                                                                                                                                                                                                                                                          `ataAGGATTGGCGTCTGGGAGGACGGAGAAGACTTACAAGATGAATAATGG‑‑‑‑‑‑‑‑‑GGG‑‑‑‑‑‑‑‑‑GAAGTC‑‑‑‑‑‑‑‑‑Gaacgacagaaagaaattgaccgagcaaaggccggtaaagaaggacaagaggggcggttagaggcaagcagagtcaaacgtcgcaaccgaaaggcacgtaaagcacacaaagctaagcgcgaaagaatgcttgctgcgtggcgatgggctgaacgtcaagaacggcgtaaacatgaggtagctgtagatgtactaggaagaaccaataacgctatgctctgggtcaacatgttctctgggg                                                <  1:211475‑M1/300‑241 (MQ=255)`  
                                                                                                                                                                                                                                                                `aTTGACCTCTGGGAAGACGCAGAAGACTTGCTCAATGAATACTTG‑‑‑‑‑‑‑‑‑GAGGAAGTCGATGAAGTC‑‑‑‑‑‑‑‑‑Gcacgacagaaagaaattgaccgcgctaaggcccgtaaagaacgtcacgaggggcgcttacatgaacgcagattcaaacgtcgcaaccgcaaggcacgtaaagcacacaaagctcagcgcgaaagaatgctttctgcgtggcgatgggctgaacgtcaagaccggcgtaaccatgaggtggctgtagatatactaggcagaaacaagcaagccattctctggttcaacatgttctctg                                                   >  1:225234‑M1/1‑64 (MQ=255)`  
                                                                                                                                                                                                                                                                 `ttGACCTCTGGGAAGACGCAGAAGACTTGCTCAATGAATACTTGGAGGAAGTCGAG‑‑‑‑‑‑‑‑‑GAAGTC‑‑‑‑‑‑‑‑‑Gcacgacagaaagaaattgaccgcgctaaggcccgtaaagaacgtcacgaggggcgcttagaggcacgcagattcaaacgtcgcaaccgcaaggcacgtaaagcacacaaagctaagcgtgaaagaatgcttgctgcgtggcgatgggctgaacgtcaagaacggcgtaaccatgaggtagctgtagatgtactaggaagaaccaataacgctatgctctgggtcaacatgttctcctg                                                  <  1:192690‑M1/301‑239 (MQ=255)`  
                                                                                                                                                                                                                                                                       `cctgGGAAGACGCAGAAGACTTGCTCAATGATTACTTG‑‑‑‑‑‑‑‑‑GAG‑‑‑‑‑‑‑‑‑GAAGTC‑‑‑‑‑‑‑‑‑Gcacgacagaaagaaattgaccgcgctaaggcccgtaaagaacgtcacgaggggcgcttagaggcacgcagattcaaacgtcgcaaccgcaaggcacgtaaagcacacaaagctaagcgcgaaagaatgcttgctgcgtggcgatgggctgaacgtcaagaacggcgtaaccatgaggtagctgtagatgtactaggaagaaccaataacgctatgctctgggtcaacatgttctctggggactttaaggcgct                                   <  1:118891‑M1/300‑254 (MQ=255)`  
                                                                                                                                                                                                                                                                        `ctGGGAAGACGCAGAAGACTTGCTCAATGAATACTTGGAGGAAGTCGAG‑‑‑‑‑‑‑‑‑GAAGTC‑‑‑‑‑‑‑‑‑Gcacgacagaaagaaattgaccgcgctaaggcccgtaaagaacgtcacgaggggcgcttagaggcacgcagattcaaacgtcgcaaccgcaaggcacgtaaagcacacaaagctaagcgcgaaagaatgcttgctgcgcggcgatgggctgaacgtcaagaacggcgtaaccatgaggtagccgtagatgtactaggaagaaccaataacgccaacccccgggccaacatgtacctgtcacttata                                           >  1:258654‑M1/1‑56 (MQ=255)`  
                                                                                                                                                                                                                                                                        `ctGGGAAGACGCAGAAGACTTGCTCAATGAATACTTG‑‑‑‑‑‑‑‑‑GAG‑‑‑‑‑‑‑‑‑GAAGTC‑‑‑‑‑‑‑‑‑Gaggaagtcacacgacagaaagaaattgaccgcgctaaggcccgtaaagaacgtcacgaggggcgcttagaggcacgcagattcaaacgtcgcaaccgcaaggcacgtaaagcacacaaagctaagcgcgaaagaatgcttgctgcgtggcgatgggctgaacgtcaagcacggcgtaaccatgatgtagctgtggatgtactaggcagaaccaataacgatatgctctgggtcaacatgttctctggagacttt                                  >  1:195681‑M1/1‑47 (MQ=255)`  
                                                                                                                                                                                                                                                                            `gAAGACGCAGAAGACTTGCTCAATGAATACTTGGAGGAAGTCGAG‑‑‑‑‑‑‑‑‑GAAGTC‑‑‑‑‑‑‑‑‑Gcacgacagaaagaaattgaccgcgctaaggcccgtaaagaacgtcacgaggggcgcttagaggcacgcagattcaaacgtcgcaaccgcaaggcacgtaaagcacacaaagctaagcgcgaaagaatgcttgctgcgtggcgatgggctgaacgtcaagaacggcgtaaccatgaggtagctgtagatgtactaggaagaaccaataacgctatgctctgggtcaacatgttctctggggactttaagg                                       <  1:238280‑M1/301‑250 (MQ=255)`  
                                                                                                                                                                                                                                                                               `gACGCAGAAGACTTGCTCAATGAATACTTGGAGGAAGTCGAG‑‑‑‑‑‑‑‑‑GAAGTC‑‑‑‑‑‑‑‑‑Gcacgacagaaagaaattgaccgcgctaaggcccgtaaagaacgtcacgaggggcgcttagaggcacgcagattcaaacgtcgcaaccgcaaggcacgtaaagcacacaaagctaagcgcgaaagaatgcttgctgcgtggcgatgggctgaacgtcaagaacggcgtaaccatgaggtagctgtagatgtactaggaagaaccaataacgctatgctctgggtaaacatgttctctagggactttaaggcgc                                    >  1:153878‑M1/1‑49 (MQ=255)`  
                                                                                                                                                                                                                                                                                   `aagaagacTTGCTCAATGAAGACTTG‑‑‑‑‑‑‑‑‑GAG‑‑‑‑‑‑‑‑‑GAAGTC‑‑‑‑‑‑‑‑‑Gcacgacagaaagaaagtgaccgcgctaaggcccgtaaagaacgtcacgaggggagcttagaggcacgcagatgcaaacgtcgcaaccgaaaggcacgtaaagcacacaaagctaagcgcgaaagaatgcttgctgcgtggcgatgggctgaacgtcaagaacggcgtaaccatgaggtagctgtagatgtactaggaagaaccaataacgctatgctctgggtcaacatgttctctggggactttaaggcgcttgaggaacgaat                       <  1:8240‑M1/300‑266 (MQ=255)`  
                                                                                                                                                                                                                                                                                     `aatgtgtataagagacagtacttg‑‑‑‑‑‑‑‑‑gag‑‑‑‑‑‑‑‑‑gaagtc‑‑‑‑‑‑‑‑‑gcacgacagaaagaaattgaccgcgctaaggcccgtaaagaacgtcacgaggggcgcttagaggcacgcagattcaaacgtcgcaaccgcaaggcacgtaaagcacacaaagctaagcgcgaaagaatgcttgctgcgtggcgatgggctgaacgtcaagaacggcgtaaccatgaggtagctgtagatgtactaggaagaaccaataacgctatgctctgggtcaacatgttctctggggactttaaggcgcttgaggaacgaatcg                     <  1:216781‑M1/283‑268 (MQ=255)`  
                                                                                                                                                                                                                                                                                                  `aTGAATACTTG‑‑‑‑‑‑‑‑‑GAG‑‑‑‑‑‑‑‑‑GAAGTC‑‑‑‑‑‑‑‑‑Gcacgacagaaagaaattgaccgcgctaaggcccgtaaagaacgtcacgaggggcgcttagaggcacgcagattcaaacgtcgcaaccgcaaggcacgtaaagcacacaaagctaagcgcgaaagaatgcttgctgcgtggcgatgggctgaacgtcaagaacggcgtaaccatgaggtagctgtagatgtactaggaagaaccaataacgctatgctctgggtcaacatgttctctggggactttaaggcgcttgaggaacgaatcgcgctgcactggcg        <  1:330043‑M1/301‑281 (MQ=255)`  
                                                                                                                                                                                                                                                                                                  `aTGAATACTTG‑‑‑‑‑‑‑‑‑GAG‑‑‑‑‑‑‑‑‑GAAGTC‑‑‑‑‑‑‑‑‑Gcacgacagaaagaaattgaccgcgctaaggcacgtaaagaacgtcacgaggggagcttagaggcacgcagattcaaacgtcgcaaccgaaaggcacgtaaagcacacaaagctaagcgcgaaagaatgcttgctgcgtggcgatgggctgaacgtcaagaacggcgtaaccatgaggtagctgtagatgtactaggaagaaccaataacgctatgctctgggtcaacatgttctctggggactttaaggagcttgaggaacgaatcgcgctgcactggcg        >  1:295024‑M1/1‑21 (MQ=255)`  
                                                                                                                                                                                                                                                                                                        `gattg‑‑‑‑‑‑‑‑‑gAG‑‑‑‑‑‑‑‑‑GAAGTC‑‑‑‑‑‑‑‑‑Gcacgacagaaagaaattgaccgcgctaaggcccgtaaagaacgtcacgaggggcgcttagaggcacgcagattcaaacgtcgcaaccgcaaggcacgtaaagcacacaaagctaagcgcgaaagaatgcttgctgcgtggcgatgggctgaacgtcaagaacggcgtaaccatgaggtagatgtagatgtactaggaagaaccaataacgctatgctctgggtcaacatgttctctggggactttaaggcgcttgaggaacgaatcgcgctgcactggcgtaatgc  <  1:159562‑M1/299‑287 (MQ=255)`  

                                                                                                                                                                                                                                                                                                             `|`                                                                                                                                                                                                                                                                                                                                    
`GACTTACAACAACGTTTTCGACCACGCTTACGAAATGCTGAAAGAAAACATCCGTTATGATGACATCCGTGACACTGATGACCTGCACGATGCTATTCACATGGCTGCCGATAATGCAGTTCCGCACTACTACGCTGACATCTTTAGCGTAATGGCAAGTGAGGGCATTGACCTTGAGTTCGAAGACTCTGGTCTGATGCCTGACACCAAGGACGTAATCCGCATCCTGCAAGCGCGTATCTATGAGCAATTAACGATTGACCTCTGGGAAGACGCAGAAGACTTGCTCAATGAATACTTG‑‑‑‑‑‑‑‑‑GAG‑‑‑‑‑‑‑‑‑GAAGTC‑‑‑‑‑‑‑‑‑GAGGAGTACGAGGAGGATGAAGAGTAATGTCTACTACCAACGTGCAATACGGTCTGACCGCTCAAACTGTACTTTTCTATAGCGACATGGTGCGCTGTGGCTTTAACTGGTCACTCGCAATGGCACAGCTCAAAGAACTGTACGAAAACAACAAGGCAATAGCTTTAGAATCTGCTGAGTGATAGACTCAAGGTCGCTCCTAGCGAGTGGCCTTTATGATTATCACTTTACTTATGAGGGAGTAATGTATATGCTTACTATCGGTCTACTCACCGCTCTAGGTCTAG  >  NC_001604.1/942‑1538`

  

Alignment Legend

Aligned base mismatch/match (shaded by quality score): `ATCG/ATCG` `< 3 ≤` `ATCG/ATCG` `< 10 ≤` `ATCG/ATCG` `< 22 ≤` `ATCG/ATCG` `< 34 ≤` `ATCG/ATCG` `< 38 ≤` `ATCG/ATCG`

Unaligned base: `atcg`    Masked matching base: `atcg`    Alignment gap: `‑`    Deleted base: `‑`
